# Relationships between urinary metals concentrations and cognitive performance among U.S. older people in NHANES 2011–2014

**DOI:** 10.3389/fpubh.2022.985127

**Published:** 2022-09-06

**Authors:** Xiangdong Wang, Pei Xiao, Rui Wang, Chao Luo, Zeyao Zhang, Shali Yu, Qiyun Wu, Ye Li, Yali Zhang, Hongbing Zhang, Xinyuan Zhao

**Affiliations:** ^1^Department of Occupational Medicine and Environmental Toxicology, Nantong Key Laboratory of Environmental Toxicology, School of Public Health, Nantong University, Nantong, China; ^2^Center for Non-Communicable Disease Management, National Center for Children's Health, Beijing Children's Hospital, Capital Medical University, Beijing, China; ^3^Jiangsu Preventive Medicine Association, Nanjing, China; ^4^Department of Biochemistry and Molecular Biology, Medical School, Nantong University, Nantong, China; ^5^Jiangsu Provincial Center for Disease Control and Prevention, Nanjing, China

**Keywords:** urine metals, cognitive function, NHANES, older people, mild cognitive impairment (MCI)

## Abstract

**Background:**

Epidemiological evidence on Urine metals and cognitive impairment in older individuals is sparse and limited. The goal of this study was to analyze if there was a link between urinary metal levels and cognitive performance in U.S. people aged 60 and up.

**Methods:**

The National Health and Nutrition Examination Survey (NHANES) data from 2011 to 2014 were utilized in this cross-sectional analysis. Memory function was quantified using the following methods: Established Consortium for Word Learning in Alzheimer's Disease (CERAD-WL) (immediate learning and recall and delayed recall), Animal Fluency Test (AFT), and Digit Symbol Substitution Test (DSST). An inductively coupled plasma mass spectrometry (ICP-MS) was used to estimate urine metal concentrations. The connection of Urine metals level with cognitive function was investigated employing binary logistic regression and restricted cubic spline models.

**Results:**

A total of 840 participants aged 60 years and over were enrolled in this study. After controlling for confounders, the association between cadmium, barium, cobalt, cesium, manganese, and thallium and poor cognitive performance showed significance in multiple logistic regression compared to the lowest quartile of metals. In the DSST test, the weighted multivariate adjusted ORs (95% CI) for cadmium in the highest quartile, barium and cesium in the third quartile were 2.444 (1.310–4.560), 0.412 (0.180–0.942) and 0.440 (0.198–0.979), respectively. There were L-shaped associations between urine cesium, barium, or manganese and low cognitive performance in DSST. Urine lead, molybdenum and uranium did not show any significant relationships with cognitive impairment, respectively, compared to the respective lowest quartile concentrations.

**Conclusion:**

The levels of barium (Ba), cobalt (Co), cesium (Cs), manganese (Mn), and thallium (Tl) in urine were found to be negatively related to the prevalence of impaired cognitive performance in our cross-sectional investigation. Higher cadmium (Cd) levels were associated with cognitive impairment.

## Introduction

Cognitive function is the ability of the human brain to recognize and reflect objective things, including various abilities such as perception, attention, memory, thinking, and language. In the process of aging, the cognitive function of the elderly changes significantly, which will have a certain degree of impact on the quality of life of the elderly, and even affect their daily life in severe cases (1). How to effectively maintain the cognitive function of the elderly has become one of the focuses of public health research and attention of the elderly.

Mild cognitive impairment (MCI) is usually used to describe the phenomenon of cognitive impairment, manifested as memory impairment and/or mild impairment of other cognitive functions (2). The American Academy of Neurology (AAN) announced a revised edition of its MCI clinical recommendations in 2018, stating that the prevalence of MCI ranged from 6.7 to 25.2% in people aged 60–84, and that the prevalence increased with age (3). MCI is the most prevalent early indication of Alzheimer's disease (AD) (short-term memory loss), which seriously affects the physical and mental health of the older, poses a significant threat to their quality of life, and often leads to a series of adverse health outcomes in the older people, such as dementia, falls, disability, decreased activities of daily living, increased hospital admissions, increased medical costs, and death (4–6). Unpaid dementia care was estimated to be worth $256.7 billion in 2020. Its costs, however, extend to an increased risk of emotional distress and unfavorable mental and physical health outcomes for home carers, which is compounded by COVID-19. In 2021, it was anticipated that the entire cost of long-term care, healthcare, and hospice services for those 65 and older with dementia would be $355 billion (7).

At present, the central pathogenesis hypothesis of Alzheimer's disease is that β amyloid (Aβ) undergoes extracellular deposition and intracellular Tau protein phosphorylation under the action of various enzymes, which has a toxic effect on neurons, and activates the glial cells in the brain to produce inflammatory factors to induce an inflammatory response (8). In this process, the participation of various enzymes is required. The trace metals involved in this study, such as barium, cadmium, and cesium, may have effects not only on ion pathways, but also directly or indirectly on proteins involved in various life activities (9, 10). These proteins may be involved in DNA repair processes, prevent DNA oxidative damage, and maintain DNA methylation, thereby affecting changes in cognitive function.

Heavy metals are widely distributed in the environment and can enter the human body through various means, such as food, drinking water and air (11, 12). Due to urbanization and industrialization, emissions from metal smelters and leachate from landfills containing toxic metals can have serious impacts on the environment and human health by contaminating groundwater, soil, surface water and natural ecosystems (13, 14). The metals and their compounds covered in this study are widely used in various products. For instance, barium salts are used to make plastics (15), and the manganese industry mainly produces batteries, ceramics, steel, cosmetics, leather, fireworks and glass, and cadmium is commonly used in battery making (16). They have been linked to the development of neurological illnesses in prior research, excessive intake of heavy metals, such as mercury and manganese are neurotoxic, and promote neurodegeneration (17, 18). Furthermore, another study showed that Cd-induced neuronal death in cortical neurons is caused by a collective mechanism of apoptosis and necrosis involving the production of reactive oxygen species and lipid peroxidation (19).

In a previous study (20), based on a similar NHANES dataset, by log-transforming levels of metals and their metabolites, Sasaki et al. analyzed the relationship between levels of metals and their metabolites (including 7 kinds in blood and 19 kinds in urine) and cognitive test scores (CERAD and DSST), using linear correlation. Their results showed that urinary metal concentrations of lead, cadmium and tungsten were significantly and negatively associated with cognitive function. Although similar data sets were used in this study, different statistical inference methods were used, and the original data were corrected, resulting in different statistical results. Moreover, besides CERAD and DSST, we also included the AFT score as an outcome variable in the statistical model. This is a more comprehensive assessment of cognitive function and selected different potential confounders, such as body mass index (BMI) and poverty-income ratio (PIR), based on previous studies (21, 22).

We analyzed the NHANES dataset from 2011 to 2014, with data from non-institutionalized U.S. civilians aged 60 and older, to look into the association between urinary metal levels and cognitive impairment, providing a new perspective for studying the pathological mechanism of MCI.

## Methods

### Study population

**Figure 1:**
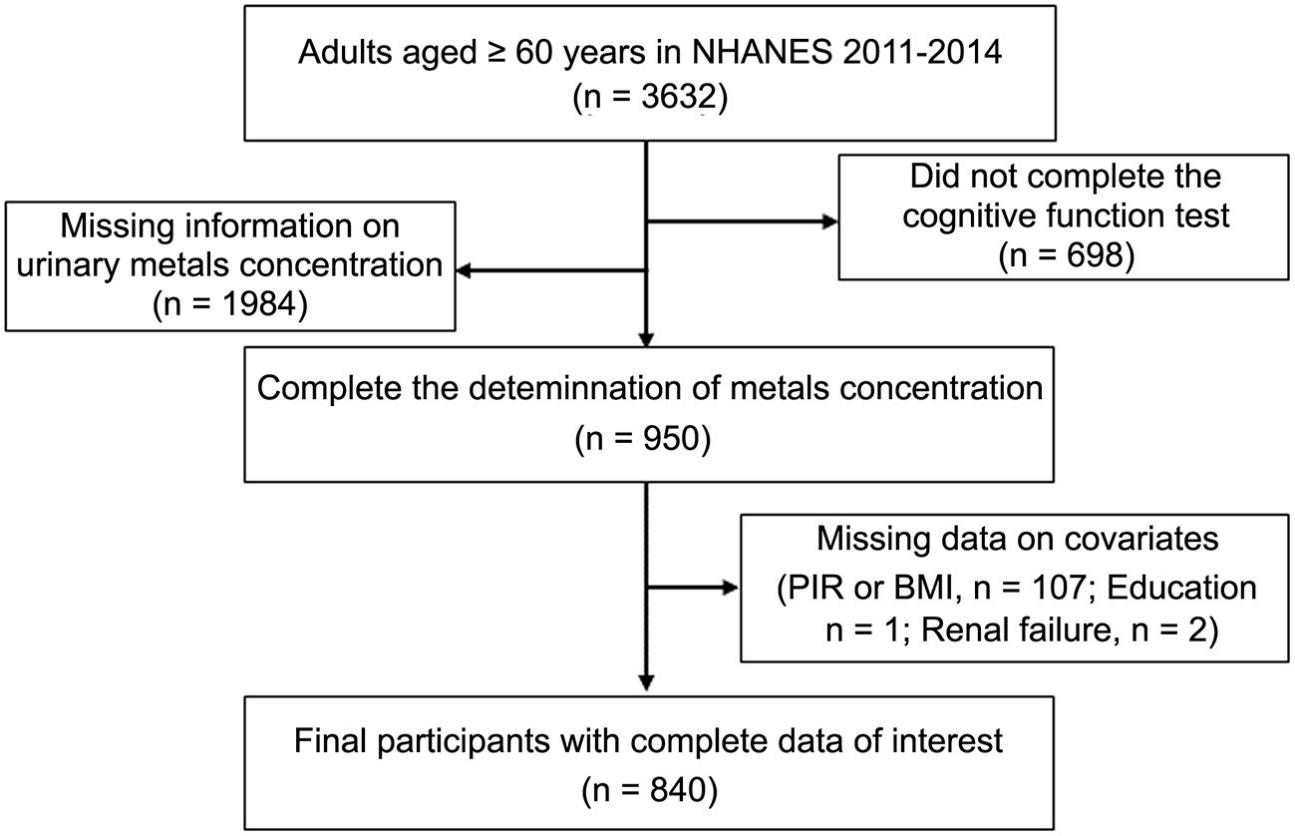
Flow chart of the selection of eligible subjects.

NHANES was conducted biannually in the United States from 1999 onwards, using a stratified multistage probability cluster design to provide a representative sample of the civilian, non-institutionalized U.S. population (23). Two cycles of NHANES (2011–2012 and 2013–2014) were included in this study because Cognitive Function Tests were measured in those cycles. Participants with missing data on urinary metal levels, cognitive tests, and covariates were excluded from the analysis. During this inclusion screening process, for age, 3,632 participants were available for analysis. We excluded 1,984 and 698 participants when examining Urine metal concentration and cognitive tests, respectively, due to the lack of completion of relevant tests. In the final research population, we assessed data from 840 adults aged 60 years and older (Figure 1). NHANES was performed by the Centers for Disease Control and Prevention (CDC), the National Center for Health Statistics (NCHS) Ethics Review Board authorized the study, and all participants gave their informed permission.

### Cognitive performance test

NHANES uses the following methods to assess memory function: the Consortium to Establish a Registry for Alzheimer's Disease Word Learning (CERAD-WL) (immediate learning and recall and delayed recall), Animal Fluency Test (AFT), and Digital Symbol Substitution Test (DSST). Cognitive function data was retrieved from the Cognitive Function Questionnaire to assess the working memory/executive, semantic memory, and episodic memory function of study subjects.

CERAD-WL is a high-reliability and validity cognitive function evaluation scale that consists of three learning trials, alone with a delay trial. Participants were instructed to read aloud ten random and unrelated words, which are displayed on a monitor one at a time after visually or auditorily showing the participant ten words, then recall as many as possible immediately. After three rounds of testing, total scores for immediate learning and recall (range: 0–30) were calculated by summing the number of correct answers per round by each participant. Delayed trials begin ~8–10 min after the first round of immediate recall trial (Tests are usually carried out after AFT and DSST are completed). Participants were required to call to mind as many of the ten words as they could. The CERAD-Total was calculated by adding the scores from the three immediate trials and one delayed trial, with a maximum score of 40 (24, 25).

Verbal Fluency Test (VFT) is a neuropsychological testing method, which involves the patient's memory, language (including naming, comprehension, semantic knowledge, etc.), execution and other cognitive functions during its operation. Animal Verbal Fluency Test is a simple method to measure semantic fluency in VFT, this test often used in clinical and research. The patient was asked to say as many names of animals as they can in 1 min, with each listed animal worth one score (24, 25).

Develop the Digit Sign Substitution Test as an experimental tool for understanding human associative learning. The DSST is currently one of the most regularly used tests in clinical neuropsychology to evaluate working memory, processing speed, and sustained attention due to its simplicity and excellent discriminant validity. Participants were given an examination paper with 9 numbers at the top coupled with symbols that functioned as a key, an array of 133 numbers below the key, and they were required to draw the proper symbol next to each number as soon as they could in 2 min. A point is awarded for each correct match, up to a maximum of 133 points (24, 25).

For CERAD, animal fluency, and DSST outcomes, there is currently no gold standard cut-off threshold; however, prior research (26) utilizing the NHANES database identified persons in the study group with the lowest score in the quartile as cognitively impaired. As a result, the 25th percentile was chosen as the threshold for poor cognitive function in this study. Participants with a CERAD-Total score of 21, an AFT score of 13, and a DSST score of 33 were characterized as having impaired cognitive function.

### Measurement of urinary metals levels

After confirming that there was no background contamination in the collected material, the research team collected a urine sample for each respondent at the sampling site. At 4°C transport environment, these samples were transported to the Division of Laboratory Science at the US Centers for Disease Control and Prevention in Atlanta, Georgia. Inductively coupled plasma mass spectrometry (ICP-MS) was used to analyze all urine samples in this investigation for metals. ICP-MS is a multi-element analysis technology that may be paired with Dynamic Reaction Cell (DRC) for trace element analysis (27, 28). In this research, we used ICP-MS for the determine the levels of cadmium (U-Cd), lead (U-Pb), barium (U-Ba), cobalt (U-Co), cesium (U-Cs), manganese (U-Mn), molybdenum (U-Mo), thallium (U-Tl) and uranium (U-Ur) in urine. If the urine metal content was below the limit of detection (LOD), the value for this variable was the limit of detection divided by the square root of two (27, 28). Metal concentrations were standardized by urine creatinine and presented as μg/g creatinine when accounted for changes in urine dilution in spot urine samples between participants.

### Covariates

The cause of cognitive impairment has been intensively researched over the last several decades. Various personal and environmental variables, such as age, PIR, education, obesity, and so on, have been linked to poor cognitive function. To control for the effect of confounders on the study results, the statistical model was adjusted for covariates to minimize any potential confounding bias caused by these factors. Covariates, such as demographic characteristics [age (60–70, 70–80, and ≥80 years); race (Mexican American, non-Hispanic white, non-Hispanic black, other Hispanic and other race); sex (male and female); marital status (married/living with partner and widowed/divorced/separated/never married) and educational level (less than high school, high school, and higher than high school)], lifestyle [smoking status (current, ever, never); BMI (normal: <25 kg/m^2^, overweight: 25 to <30 kg/m^2^, obese: ≥30 kg/m^2^) and PIR (under poverty level: ≤ 1, above poverty level: >1) (29)], and questionnaire findings (Self-reported physician diagnosis of hypertension, renal failure and diabetes is considered to be a history of hypertension, renal failure and diabetes), were gathered by uniform interviews, physical and laboratory testing, and questionnaires administered by competent medical personnel.

### Statistical analysis

Since NHANES employs a probability sampling strategy, we selected a 2-year weight (wtsa2yr) for the subsamples for urine metal concentration testing in NHANES 2011–2012 and 2013–2014 according to the analysis guidelines of the NHANES database. When the two survey cycles were joined, the ultimate weight (1/2 wtsa2yr for NHANES 11-14) was adjusted (30).

Descriptive statistics for variables, Chi-square tests were chosen to compare the categorical variables that were represented by numerical and frequency distribution, including age, race, sex, education levels, alcohol consumption, marital status, history of hypertension, diabetes and renal failure. The lowest quartile (Q1) was used as the reference group for each urine metal, then we fitted the multivariate logistics regression to evaluate the effect of a single urinary metal on cognitive function by comparing Q2, Q3, Q4 to Q1 and to generate odds ratios (ORs) and 95% confidence intervals (CIs) to evaluate the prevalence of urine metals-related cognitive impairment. Based on previous research and theoretical concerns, in logistic regression, different potential confounders were adjusted in Model 1 (no confounders were adjusted), Model 2 (adjustments in Model 1 plus age and sex) and Model 3 (Model 3 was the same as Model 2 with additional adjustment for BMI, education, PIR, marital status, race, smoke, renal failure, hypertension and diabetes). A restricted cubic spline regression was performed to accommodate the non-linearity of the association between urinary metal concentrations and cognitive function, using four nodes in the model.

Weighted and RCS analyses were operated with the “survey” and “rms” package, respectively, in the R software (version 4.1.1, R Foundation for Statistical Computing). All *p*-values are two-tailed, with 0.05 chosen as the statistical significance level.

## Results

Four hundred seventeen males and 423 females from NHANES (2011–2014) participated in our research. Respondents' sociodemographic data were described and categorized by sex, age, education level, ethnicity, marital status, smoking habits, PIR and BMI. There were significant differences in the distributions of age, ethnicity, education, hypertension, and PIR between those with poor and normal cognitive performance for all three measures of cognitive function. In contrast, The BMI of those with normal cognitive performance and those with low cognitive performance did not differ significantly, according to Chi-square testing. Diabetes, renal failure, and hypertension were more common in adults with poor cognitive performance than in people with normal cognitive performance. We made statistical inferences about whether there were differences in the distribution of basic characteristics between the three groups of cognitively impaired populations, based on three different tests. We observed significant differences in the distribution of gender, race, and educational attainment among the three groups. Table 1 and Supplementary Table S1 provides detailed baseline data for this investigation. Geometric means, quartiles and detection rates of urine metals were presented in Table 2. The median concentrations and geometric means of the 10 metals in urine were ranged from 0.0065 to 39.2500 μg/g creatinine, and 0.0072–39.6731 μg/g creatinine, respectively.

**Table 1:** Characteristics of the study population, National Health and Nutrition Examination Survey (NHANES) 2011–2014 (*N* = 840).

	**CERAD test**			**Animal fluency test**			**Digit symbol test**		
**Catalogs**	**Normal**	**Low**	***p*-value**	**Normal**	**Low**	***p*-value**	**Normal**	**Low**	***p*-value**
	**Cognitive**	**Cognitive**		**Cognitive**	**Cognitive**		**Cognitive**	**Cognitive**	
	**Performance**	**Performance**		**Performance**	**Performance**		**Performance**	**Performance**	
**Number of subjects (%)**	633	207		640	200		642	198	
**Age (%)**			<0.01			<0.01			<0.01
60–70 years	375 (59.24)	83 (40.10)		376 (58.75)	82 (41.00)		373 (58.10)	85 (42.93)	
70–80 years	200 (31.60)	63 (30.43)		192 (30.00)	71 (35.50)		192 (29.91)	71 (35.86)	
≥80 years	58 (9.16)	61 (29.47)		72 (11.25)	47 (23.50)		77 (11.99)	42 (21.21)	
**Sex (%)**			<0.01			0.990			<0.01
Male	280 (44.23)	137 (66.18)		318 (49.69)	99 (49.50)		296 (46.11)	121 (61.11)	
Female	353 (55.77)	70 (33.82)		322 (50.31)	101 (50.50)		346 (53.89)	77 (38.89)	
**Race (%)**			0.049			<0.01			<0.01
Mexican American	45 (7.11)	24 (11.60)		49 (7.66)	20 (10.00)		35 (5.45)	34 (17.18)	
Other Hispanic	54 (8.53)	27 (13.04)		62 (9.69)	19 (9.50)		50 (7.79)	31 (15.66)	
Non-Hispanic White	312 (49.29)	95 (45.89)		332 (51.88)	75 (37.50)		345 (53.74)	62 (31.31)	
Non-Hispanic Black	161 (25.43)	41 (19.81)		137 (21.41)	65 (32.5)		138 (21.49)	64 (32.81)	
Other race	61 (9.64)	20 (9.66)		60 (9.38)	21 (10.5)		74 (11.53)	7 (3.54)	
**Educational level (%)**			<0.01			<0.01			<0.01
Below high school	126 (19.91)	86 (41.55)		141 (22.03)	71 (35.50)		101 (15.74)	111 (56.06)	
High school	143 (22.59)	43 (20.77)		129 (20.16)	57 (28.50)		143 (22.27)	43 (21.72)	
Above high school	364 (57.50)	78 (37.68)		370 (57.81)	72 (36.00)		398 (61.99)	44 (22.22)	
**Marital status (%)**			0.744			0.014			<0.01
Married/living with partner	369 (58.29)	124 (59.90)		391 (61.09)	102 (51.00)		396 (61.68)	97 (48.99)	
Widowed/divorced/separated/never married	264 (41.71)	83 (40.10)		249 (38.91)	98 (49.00)		246 (38.32)	101 (51.01)	
**Poverty–income ratio (%)**			0.025			<0.01			<0.01
≤ 1	112 (17.69)	52 (25.12)		106 (16.56)	58 (29.00)		96 (14.95)	68 (34.34)	
>1	521 (82.31)	155 (74.88)		534 (83.44)	142 (71.00)		546 (85.05)	130 (65.66)	
**Body mass index (%)**			0.121			0.524			0.337
<25 kg/m^2^	164 (25.91)	61 (29.47)		166 (25.94)	59 (29.50)		177 (28.04)	43 (22.73)	
25–30 kg/m^2^	223 (35.23)	82 (39.61)		238 (37.19)	67 (33.50)		227 (35.67)	75 (38.38)	
≥30 kg/m^2^	246 (38.86)	64 (30.92)		236 (36.87)	74 (37.00)		227 (36.29)	74 (38.89)	
**Smoking status (%)**			0.362			0.704			0.036
Never	309 (48.82)	101 (48.79)		309 (48.28)	101 (50.50)		318 (49.53)	92 (46.46)	
Former	251 (39.65)	89 (43.00)		264 (41.25)	76 (38.00)		265 (41.28)	75 (37.88)	
Current	73 (11.53)	17 (8.21)		67 (10.47)	23 (11.50)		59 (9.19)	31 (15.66)	
**Hypertension (%)**	385 (60.82)	141 (68.12)	0.072	373 (58.44)	152 (76.00)	<0.01	388 (60.44)	138 (69.70)	0.023
**Diabetes (%)**	137 (21.64)	52 (25.12)	0.345	127 (19.84)	62 (31.00)	<0.01	127 (19.78)	62 (31.31)	<0.01
**Renal failure (%)**	34 (5.37)	18 (8.70)	0.12	34 (5.31)	18 (9.00)	0.085	28 (4.36)	24 (12.12)	<0.01

*p*-value was tested by Chi-square test or Fisher's exact.

**Table 2:** Geometric means, quartiles and detection rates of urine metals.

**Urine metals (μg/g creatinine)**	**GM (95% CI)^a^**	**Median (IQR)^b^**	**Detection rate (%)^c^**
Ba	1.0749 (1.0013, 1.1539)	1.0833 (0.5375, 2.2100)	99.76
Cd	0.3432 (0.3255, 0.3618)	0.3435 (0.2039, 0.5733)	96.07
Co	0.3889 (0.3702, 0.4087)	0.3517 (0.2431, 0.5539)	99.76
Cs	4.7195 (4.5599, 4.8847)	4.6490 (3.4080, 6.4410)	100
Mo	39.6731 (37.8596, 41.5734)	39.2500 (26.4600, 59.5000)	100
Mn	0.1413 (0.1336, 0.1494)	0.1317 (0.0808, 0.2302)	46.07
Pb	0.5237 (0.4997, 0.5489)	0.5138 (0.3358, 0.7857)	98.93
Sn	0.8660 (0.8068, 0.9295)	0.7457 (0.4174, 1.7333)	92.5
Tl	0.1532 (0.1470, 0.1598)	0.1546 (0.1026, 0.2294)	99.4
Ur	0.0072 (0.0068, 0.0077)	0.0065 (0.0038, 0.0122)	78.33

^a^ G-Mean (95%).

^b^ Median (25th, 75th percentiles).

^c^ Proportion above the lower limit of detection (LLOD, in μg/L).

The relationship between urinary metal concentration and cognitive function is outlined in Table 3 and Supplementary Table S2. The Table 3 presented the associations between U-Ba, U-Co, U-Tl, U-Mn, U-Cs and U-Cd concentration and low cognitive performance as judged by different measures. After controlling for confounders, the association between cadmium and thallium in the third quartile and low cognitive performance in CERAD showed significance in multiple logistic regression compared to the lowest quartile of metals. The ORs (95% CIs), respectively, were 2.407 (1.137–5.099) and 0.509 (0.277–0.936). In the crude Model (Model 1) and adjusting for Model (Model 2 and Model 3) of Animal Fluency Test, the third quartiles (Q3) and the highest quartile (Q4) of U–Cd were linked to an increased prevalence of cognitive impairment. Our data also revealed that the relationship between U–Co and low cognitive function was significant in the crude model, with the crude OR and 95% CIs of low cognitive performance indicating that U–Co was associated with low cognitive performance in the DSST and AFT. After adjusting for age and sex, compared to the lowest quartile of U–Co, the weighted multivariate adjusted ORs (95% CI) of the third quartile were 0.454 (0.232–0.886) and 0.464 (0.260–0.829). In the DSST test, cadmium in the highest quartile, barium and cesium in the third quartile, and cobalt in the second quartile, the OR (95% CI) of the logistic regression showed significance in each model.

**Table 3:** Weighted odds ratios (95% confidence intervals) of low cognitive performance by quartiles of metals level, NHANES 2011–2014.

	**CERAD test**			**Animal fluency test**			**DSST**		
**Group**	**Model1**	**Model2**	**Model3**	**Model1**	**Model2**	**Model3**	**Model1**	**Model2**	**Model3**
	**Urine-Ba (μg/g creatinine)**								
Q1 (<0.5375)	Reference	Reference	Reference	Reference	Reference	Reference	Reference	Reference	Reference
Q2 (≥0.5375 and <1.0833)	0.699 (0.364–1.339)	0.884 (0.474–1.650)	1.212 (0.601–2.444)	**0.499 (0.278–0.893)**	0.568 (0.321–1.003)	0.790 (0.417–1.498)	0.574 (0.326–1.011)	0.668 (0.395–1.134)	1.023 (0.550–1.901)
Q3 (≥1.0833 and <2.21)	**0.405 (0.209–0.784)**	0.559 (0.284–1.103)	0.768 (0.359–1.646)	**0.250 (0.124–0.505)**	**0.279 (0.135–0.580)**	0.408 (0.164–1.016)	**0.248 (0.123–0.497)**	**0.297 (0.150–0.586)**	**0.412 (0.180–0.942)**
Q4 (≥2.21)	0.838 (0.389–1.805)	1.052 (0.518–2.134)	1.662 (0.720–3.836)	0.624 (0.358–1.088)	0.631 (0.371–1.072)	1.031 (0.510–2.083)	**0.393 (0.191–0.811)**	**0.414 (0.212–0.808)**	0.728 (0.314–1.686)
	**Urine-Cd (μg/g creatinine)**								
Q1 (<0.20391)	Reference	Reference	Reference	Reference	Reference	Reference	Reference	Reference	Reference
Q2 (≥0.20391 and <0.34352)	0.957 (0.594–1.542)	1.037 (0.567–1.897)	1.065 (0.588–1.932)	1.396 (0.783–2.49)	1.376 (0.746–2.540)	1.463 (0.806–2.655)	1.242 (0.675–2.287)	1.295 (0.685–2.448)	1.335 (0.630–2.831)
Q3 (≥0.34352 and <0.5733)	**1.993 (1.154–3.441)**	**2.187 (1.137–4.204)**	**2.407 (1.137–5.099)**	**2.074 (1.22–3.524)**	**1.844 (1.168–2.912)**	**2.016 (1.233–3.297)**	1.631 (0.777–3.423)	1.570 (0.797–3.093)	1.720 (0.777–3.806)
Q4 (≥0.5733)	1.398 (0.777–2.516)	1.634 (0.816–3.270)	1.818 (0.710–4.657)	**2.33 (1.557–3.487)**	**2.211 (1.445–3.384)**	**2.384 (1.349–4.215)**	**1.875 (1.063–3.308)**	**1.974 (1.160–3.358)**	**2.444 (1.310–4.560)**
	**Urine-Co (μg/g creatinine)**								
Q1 (<0.2431)	Reference	Reference	Reference	Reference	Reference	Reference	Reference	Reference	Reference
Q2 (≥0.2431 and <0.3517)	1.159 (0.598–2.247)	1.118 (0.568–2.198)	1.274 (0.639–2.539)	**0.524 (0.278–0.987)**	**0.454 (0.232–0.886)**	0.485 (0.218–1.079)	**0.524 (0.297–0.924)**	**0.464 (0.260–0.829)**	**0.461 (0.227–0.937)**
Q3 (≥0.3517 and <0.5539)	0.519 (0.209–1.289)	0.582 (0.244–1.388)	0.611 (0.241–1.702)	0.904 (0.483–1.689)	0.889 (0.466–1.694)	1.013 (0.486–2.110)	0.599 (0.318–1.130)	0.623 (0.339–1.142)	0.718 (0.329–1.569)
Q4 (≥0.5539)	0.835 (0.456–1.532)	0.792 (0.421–1.490)	0.899 (0.456–1.771)	0.746 (0.419–1.327)	0.582 (0.328–1.032)	0.676 (0.317–1.440)	0.518 (0.256–1.051)	**0.440 (0.207–0.935)**	0.542 (0.219–1.341)
	**Urine-Cs (μg/g creatinine)**								
Q1 (<3.408)	Reference	Reference	Reference	Reference	Reference	Reference	Reference	Reference	Reference
Q2 (≥3.408 and <4.649)	0.841 (0.433–1.634)	0.753 (0.391–1.453)	0.775 (0.368–1.634)	0.804 (0.500–1.293)	0.703 (0.451–1.096)	0.803 (0.497–1.297)	0.751 (0.403–1.399)	0.674 (0.389–1.169)	0.823 (0.458–1.479)
Q3 (≥4.649 and <6.441)	0.724 (0.395–1.326)	0.701 (0.385–1.276)	0.773 (0.387–1.546)	0.657 (0.394–1.094)	0.609 (0.359–1.032)	0.813 (0.451–1.466)	**0.397 (0.197–0.800)**	**0.370 (0.201–0.679)**	**0.440 (0.198–0.979)**
Q4 (≥6.441)	0.646 (0.319–1.308)	0.772 (0.372–1.601)	0.947 (0.410–2.189)	0.612 (0.338–1.109)	0.613 (0.351–1.072)	0.861 (0.469–1.582)	**0.318 (0.187–0.540)**	**0.332 (0.195–0.567)**	0.492 (0.240–1.009)
	**Urine-Mn (μg/g creatinine)**								
Q1 (<0.08078)	Reference	Reference	Reference	Reference	Reference	Reference	Reference	Reference	Reference
Q2 (≥0.08078 and <0.13165)	0.810 (0.484–1.354)	1.189 (0.606–2.333)	1.239 (0.629–2.442)	0.870 (0.497–1.524)	1.031 (0.571–1.860)	1.084 (0.557–2.110)	0.958 (0.559–1.643)	1.201 (0.676–2.133)	1.369 (0.750–2.497)
Q3 (≥0.13165 and <0.23019)	0.684 (0.384–1.220)	0.802 (0.411–1.565)	0.820 (0.401–1.758)	0.944 (0.574–1.551)	0.902 (0.530–1.535)	0.988 (0.541–1.806)	0.821 (0.381–1.770)	0.851 (0.405–1.787)	0.982 (0.473–2.040)
Q4 (≥0.23019)	0.588 (0.326–1.060)	1.039 (0.466–2.315)	1.093 (0.499–2.396)	0.848 (0.489–1.473)	0.981 (0.529–1.817)	1.141 (0.544–2.397)	**0.442 (0.234–0.837)**	0.564 (0.298–1.069)	0.637 (0.267–1.520)
	**Urine-Tl (μg/g creatinine)**								
Q1 (<0.1026)	Reference	Reference	Reference	Reference	Reference	Reference	Reference	Reference	Reference
Q2 (≥0.1026 and <0.15455)	0.930 (0.598–1.446)	0.886 (0.506–1.552)	0.956 (0.520–1.758)	0.683 (0.360–1.294)	0.648 (0.321–1.307)	0.679 (0.308–1.497)	0.702 (0.386–1.276)	0.673 (0.365–1.243)	0.739 (0.375–1.457)
Q3 (≥0.15455 and <0.22936)	**0.424 (0.231–0.780)**	**0.464 (0.258–0.835)**	**0.509 (0.277–0.936)**	**0.475 (0.243–0.926)**	**0.474 (0.242–0.929)**	0.582 (0.277–1.225)	0.570 (0.296–1.100)	0.618 (0.317–1.203)	0.958 (0.447–2.053)
Q4 (≥0.22936)	**0.428 (0.245–0.746)**	0.562 (0.313–1.010)	0.662 (0.387–1.133)	**0.546 (0.303–0.985)**	0.576 (0.328–1.011)	0.720 (0.382–1.357)	**0.395 (0.202–0.772)**	**0.462 (0.229–0.935)**	0.773 (0.314–1.828)

Crude model (Model 1) did not adjust any confounders. Model 2 adjusted for age (years), gender. Model 3 was the same as Model 2 with additional adjustment for educational level (less than high school, high school, higher than high school), marital status (married, widowed, divorced, separated, never married, living with partner), BMI, PIR, smoke, race, renal failure (Yes or No), hypertension (Yes or No) and diabetes (Yes or No). The bold values indicate statistically significant values of *p* <0.05.

**Figure 2:**
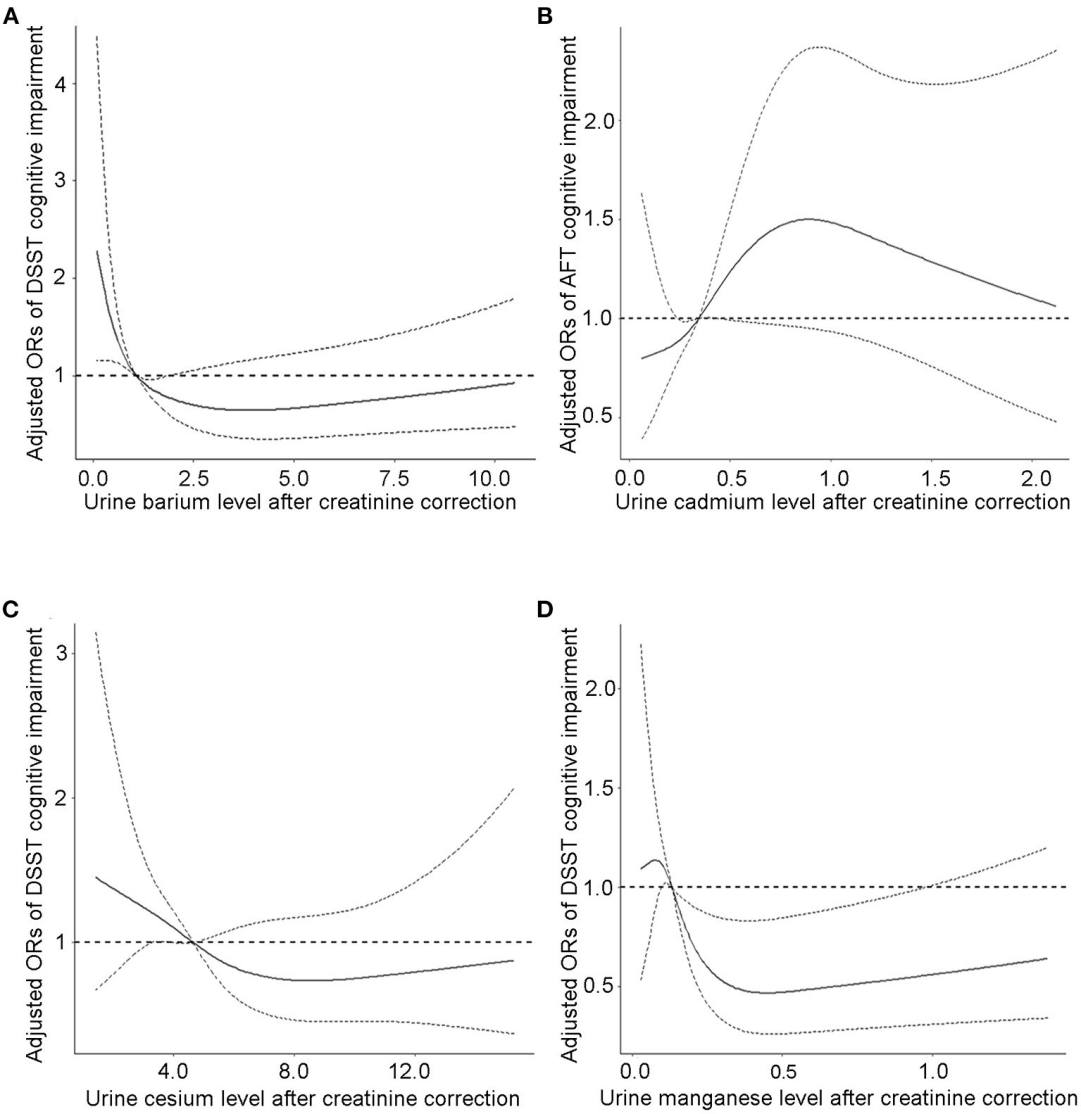
The continuous relationship between urinary **(A)** barium levels, **(C)** cesium levels, **(D)** manganese levels and DSST cognitive test scores based on a restricted cubic spline regression model. The continuous relationship between **(B)** cadmium levels and AFT cognitive test scores based on a restricted cubic spline regression model. Adjusted for covariates (age, sex, educational level, BMI, and PIR). The solid lines represent the ORs, and dashed lines represent the 95% CIs.

Analysis of the continuous relationship between urinary metal levels and cognitive test scores based on a restricted cubic spline regression model (Figure 2; Supplementary Figure S1). We discovered L-shaped associations between test scores and creatinine-adjusted urine cesium and manganese for cognitive performance in the DSST. The prevalence of low cognitive performance decreased with increasing urinary Ba, Cs, and Mn levels, whereas it increased with urinary Cd concentration, and showed a non-linear dose-response relationship. The association between CERAD cognitive impairment and urinary Cd, Mn, Cs, and Ba concentrations was insignificant (Supplementary Figures S2, S3).

## Discussion

In this large sample, NHANES-based, cross-sectional study of U.S. participants aged 60 years or older, the association between urinary metals and cognitive function was analyzed with three measures of cognitive performance. Although Sasaki and Carpenter (20), also used this dataset to illustrate the correlation between metal concentrations and cognitive scores, however, we varied widely in the selection of study subjects for inclusion, and corrected the original data. The detailed changes are listed below. Firstly, we corrected the urine metal content using urine creatinine, which was used to exclude the effect of urine concentration or dilution on the metal content. In addition, we transformed cognitive test scores into a binary variable, normal cognitive status vs. low cognitive performance. Trends in the prevalence of cognitive impairment at different urine metal levels were explored using logistic regression with restricted cubic spline models. Secondly, additional covariates were chosen in this study on the grounds that the results of Momtaz et al. support a significant association between BMI and cognitive function in the older population (22) and that higher cognitive ability is associated with increased household income (21). This allows for better control for confounding factors in the study results. Finally, to treat all participants equally, Sasaki and Carpenter performed an unweighted analysis. Our research team weighted the raw data to account for complex survey design (including oversampling), survey non-response and post-stratification before statistical extrapolation for the purpose of making the estimates representative of the non-institutionalized civilian population in the United States (30).

Many investigations have concluded that metals have a role in nervous system disorders (31). However, as far as we know, analyses focused on urine cesium exposure levels and poor cognitive performance were rare, and only a few studies have looked at the link between cesium levels in the cerebrospinal fluid and cognitive function or Alzheimer's disease (32). More recently, Almulla et al. conducted a case-control study of 120 adults with schizophrenia and 54 healthy controls, and measured cesium and cognitive impairments [using the Brief Assessment of Cognition in Schizophrenia (BACS)]. The findings imply that cesium was favorably linked with the results of the neurocognitive investigation (33). Our study showed that urinary cesium levels were inversely associated with the prevalence of cognitive impairment. The exact mechanism of the association between urinary cesium and low cognitive performance is unclear, but some speculations may be as follows. Cesium prevents neuronal apoptosis by inactivating glycogen synthase kinase 3 beta (GSK3b) through phosphorylation of serine 9. Furthermore, it inhibited caspase-3 activation and neuronal apoptosis in a dose-dependent manner, as well as H2O2-induced neuronal death, thereby boosting neuronal survival (34). Moreover, cesium (Cs+) prevented the apoptotic volume decrease, caspase-3 activation and cell death induced by K5 and camptothecin. It may have a role in the activation of the apoptotic volume decrease and apoptotic death of Cerebellar granule neurons (35).

The substantial association between U-Ba levels and many subdomains and cognitive impairment, such as immediate and delayed learning capacity, categorical linguistic fluency, episodic memory, and attention, is another noteworthy conclusion of this study. These findings also point to barium as a possible contributor to cognitive function. Gu et al. carried out a baseline survey of the Elderly Health and Environment Risk Factor Cohort, and reported that trace elements Ba could be a protective factor for cognitive function (36). This conclusion is in line with the view of our research. However, there are also other findings that blood barium may not be associated with cognitive function when concomitant exposure to other metals is considered (37). There are several possible explanations for different conclusions stated above. Due to the deficient concentration of Ba in the human body and the significant influence of external factors, statistical results cannot correctly reflect the truth, different types of samples (the metals samples in blood and urine are not the same), cognitive function testing methods are inconsistent, and so on. It's unknown what role Ba plays in cognitive function; however, one study (38) suggests that serum Ba may be associated with increased glutathione reductase (GR) activity, which protects the brain from damage.

Manganese is one of the essential trace elements in the human body and participates in forming various proteins and physiological functions. Previous studies have not given a clear answer on whether manganese exposure can lead to impaired cognitive function in humans. Altered manganese status in the body is associated with changes in human neuronal physiology and cognition, either overexposure or underexposure leading to neurological dysfunction (39). One result from a meta-analysis showed that serum manganese levels in AD patients were lower than those in controls (40). However, one study on 40 older people in China showed that high manganese level may be a causative factor for AD (41). Manganese may cause glutamate accumulation by damaging glutamate transport and reducing glutamine synthetase activity, and ultimately lead to neurotoxicity (42, 43). We found that optimum exposure to manganese may be a protective factor for cognitive dysfunction, which can be explained by the physiological function of manganese. Superoxide dismutase 2 (SOD2) with manganese as a prosthetic group is mainly distributed in mitochondria, SOD2 has antioxidant function and can reduce oxidative stress, amyloid deposition, and memory deficits in AD transgenic mouse models (44).

Cadmium, a carcinogenic heavy metal, can not only cause a variety of diseases, but also enter the brain and lead to neurological damage. An analysis of 2011–2014 data from the NHANES database showed an inverse relationship between blood cadmium and cognitive function in older adults over 60 years of age (45). By using the relevant data from the NHANES database, Min et al. found that there is a correlation between the blood cadmium level and the incidence of AD (46). Furthermore, Peng et al. provided updated evidence to support the association between cadmium and AD mortality (47). These publications echo our findings that urinary cadmium levels are associated with cognitive impairment. Both blood cadmium and urine cadmium can be employed as biomarkers for researching the relationship between body cadmium content and outcome variables, because both can accurately reflect the degree of cadmium burden in the body in terms of long-term consequences (48). The mechanism by which cadmium affects the central nervous system is still unclear. The mainstream view is that cadmium may induce neuronal apoptosis by inducing activation of astrocytes and secretion of inflammatory mediators (49, 50).

Our study had several benefits, besides the sophisticated NHANES urine metal concentration measurement technique. A major strength is that our sample of older adults is derived from the nationally representative NHANES database, which is known for its high-quality survey methodologies and quality control. In addition, considering the potential bias, we selected the main confounders in terms of lifestyle and physical condition based on previous findings to adjust the regression model, avoiding the interference of covariates to a certain extent. Besides, NHANES collected performance data on well-studied cognitive tests in different domains, although not comprehensive in scope, it may be a valuable indicator of underlying brain pathology and therefore worth investigating.

Potential limitations of this study are, first, that this is a cross-sectional design that cannot determine the temporal sequence of urinary metal exposure and cognitive function, is not suitable for examining the prospective relationship between urinary metal exposure and cognitive function. There are many unmeasured confounders from diet, environment, and lifestyle influenced the findings, so we were unable to assess causality for the association between urinary metal concentrations and low cognitive performance. Second, single-examination urinary metal concentrations may not be ideal biomarkers of exposure and adjusted creatinine concentrations may lead to variability and unexpected bias in the population, as some urinary metals reflect only short-term exposure, thus our findings should be carefully considered. Finally, since our subjects were required to be 60 years old and above, NHANES had data for cognitive tests only in 2011–2014, and the numerical values of the covariates were missing, ultimately including only 840 subjects, which may lead to biased research results.

## Conclusion

We utilized logistic regression and restricted cubic spline models to perform statistical inference on NHANES data to assess the relationship between urinary metal levels and cognitive function in healthy older persons. U-Cd showed positive associations with performance on DSST modules. Concentrations of Cesium, Manganese and Barium had negative associations with cognitive function, and the association of manganese with cognitive function remained significant even when the model was adjusted for covariates. These findings imply that Cs, Mn, Ba, and Cd may be involved in MCI pathogenesis, such as by interfering with potassium channels or protecting neurons.

## Data availability statement

Publicly available datasets were analyzed in this study. This data can be found here: the datasets for this study can be found in the NHANES repository. Please see the https://www.cdc.gov/nchs/nhanes/index.htm for more details.

## Ethics statement

The studies involving human participants were reviewed and approved by the Research Ethics Review Board (ERB) of the US National Center for Healthcare Statistics (NCHS) authorized the 2011–2014 NHANES (Protocol Number: protocol#2011-17 and continuation of protocol #2011-17). The patients/participants provided their written informed consent to participate in this study. Written informed consent was obtained from the individual(s) for the publication of any potentially identifiable images or data included in this article.

## Author contributions

XW and PX: conceptualization, methodology, software, writing—original draft, and writing—review and editing. RW, CL, and ZZ: software, visualization, data curation, and formal analysis. SY and QW: writing—review and editing. YL, HZ, and XZ: conceptualization, supervision, project administration, and writing—review and editing. YZ: revision. All authors contributed to the article and approved the submitted version.

## Funding

This work was supported by the National Natural Science Foundation of China (82173554, 82173482); Natural Science Foundation of Jiangsu Province (BK20201444); Nantong Jiangsu Scientific Research Project (JC2020042); Qing Lan Project for Excellent Young Key Teachers of Colleges and Universities of Jiangsu Province (2020); the Beijing Municipal Natural Science Foundation (7214277).

## Conflict of interest

The authors declare that the research was conducted in the absence of any commercial or financial relationships that could be construed as a potential conflict of interest.

## Publisher's note

All claims expressed in this article are solely those of the authors and do not necessarily represent those of their affiliated organizations, or those of the publisher, the editors and the reviewers. Any product that may be evaluated in this article, or claim that may be made by its manufacturer, is not guaranteed or endorsed by the publisher.
