# Supplementary material for: Relationships between urinary metals concentrations and cognitive performance among U.S. older people in NHANES 2011–2014
Source: Front Public Health. 2022 Sep 6;10:985127. doi: 10.3389/fpubh.2022.985127 (PMC9485476; doi:10.3389/fpubh.2022.985127)
Supplement: Supplementary file 1 [file Data_Sheet_1.PDF]

Supplementary Figure 1

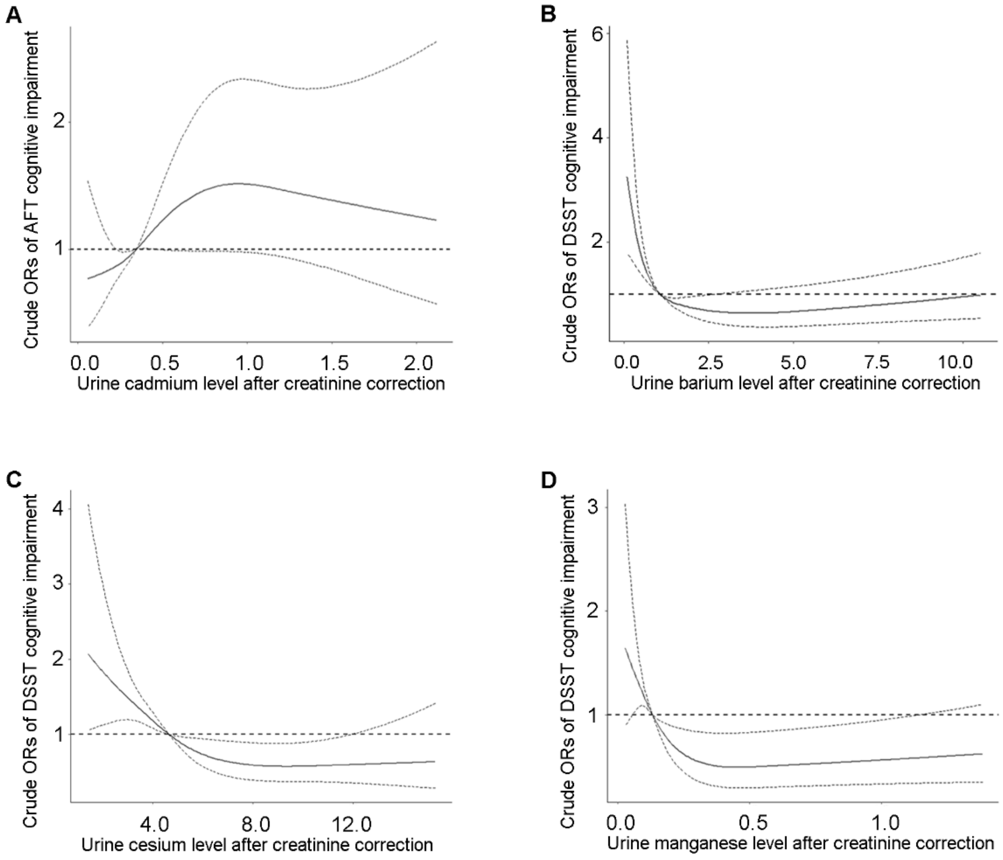

Figure. S1. The continuous relationship between urinary (B) barium levels, (C) cesium levels, (D) manganese levels and DSST cognitive test scores based on a restricted cubic spline regression model. The continuous relationship between (A) cadmium levels and AFT cognitive test scores based on a restricted cubic spline regression model.

Supplementary Figure 2

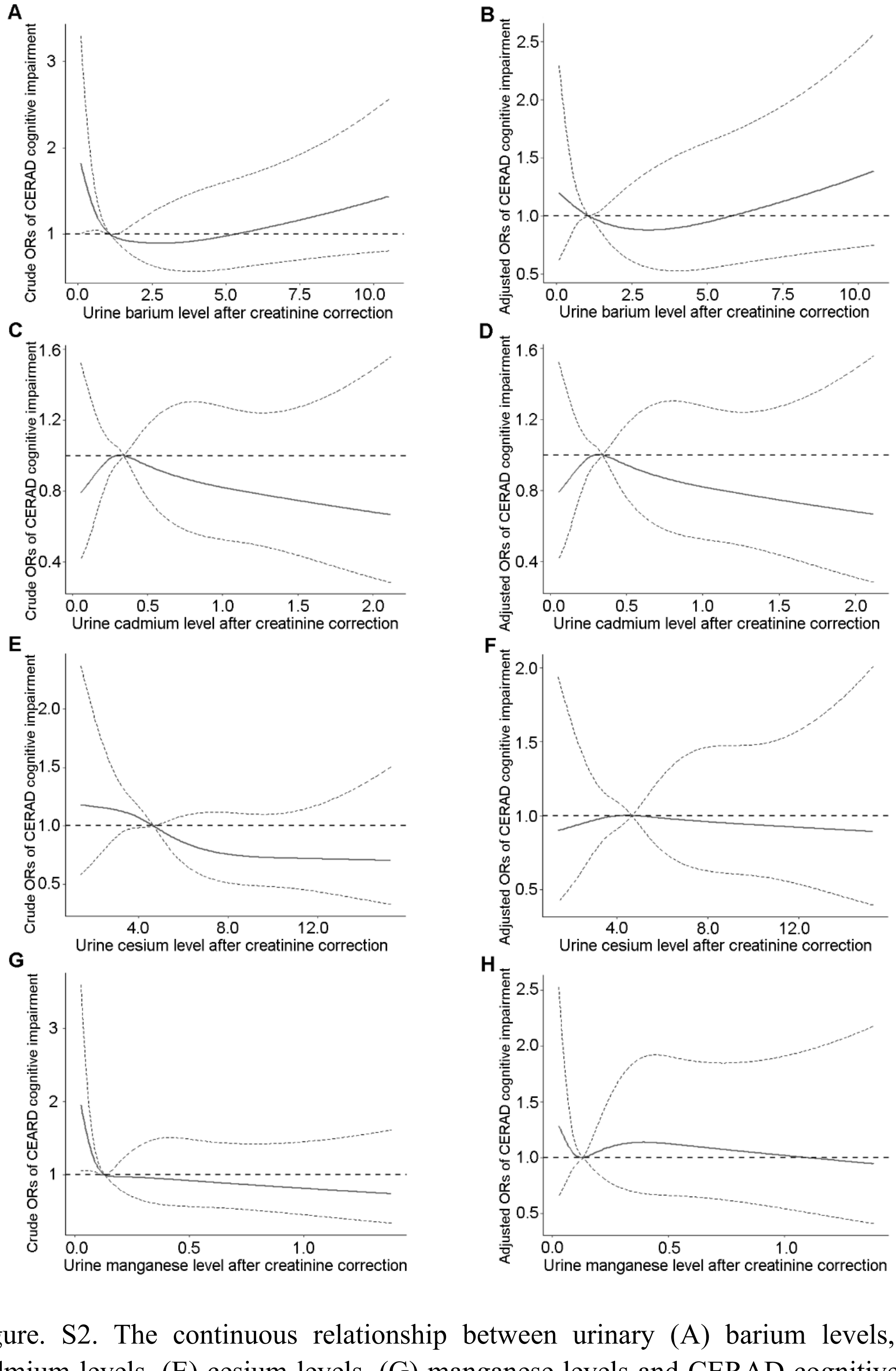

Figure. S2. The continuous relationship between urinary (A) barium levels, (C) cadmium levels, (E) cesium levels, (G) manganese levels and CERAD cognitive test scores based on a restricted cubic spline regression model. (B), (D), (F), (H) adjusted for covariates (age, gender, educational level, BMI, and poverty–income ratio). The solid lines represent the ORs, and dashed lines represent the 95% CIs.

Supplementary Figure 3

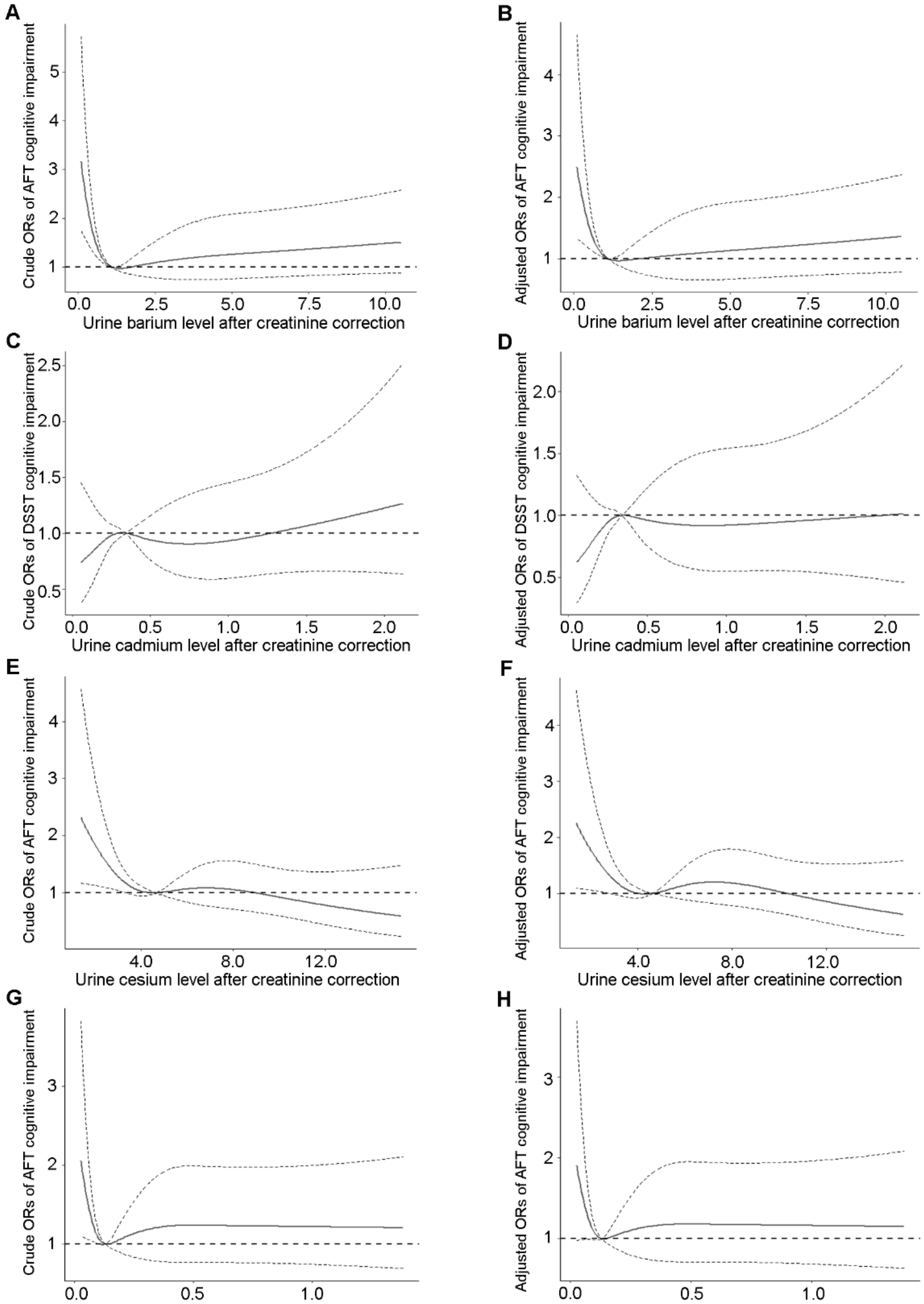

Figure. S3. The continuous relationship between urinary (A) barium levels, (E) cesium levels, (G) manganese levels and AFT cognitive test scores based on a restricted cubic spline regression model. The continuous relationship between (C) cadmium levels and DSST cognitive test scores based on a restricted cubic spline regression model. (B), (D), (F), (H) adjusted for covariates (age, gender, educational level, BMI, and poverty–income ratio.) The solid lines represent the ORs, and dashed lines represent the 95% CIs.
