# Supplementary material for: Relationships between urinary metals concentrations and cognitive performance among U.S. older people in NHANES 2011–2014
Source: Front Public Health. 2022 Sep 6;10:985127. doi: 10.3389/fpubh.2022.985127 (PMC9485476; doi:10.3389/fpubh.2022.985127)
Supplement: Supplementary file 2 [file Data_Sheet_2.PDF]

Table S1. Defined as the distribution of basic characteristics of patients with cognitive impairment

catalogues	CERAD Test Low Cognitive Performance	Animal Fluency Test Low Cognitive Performance	Digit Symbol Test Low Cognitive Performance	*p value
**Age (%)**				
60-70 years	83 (40.10)	82 (41.00)	85 (42.93)	0.367
70-80 years	63 (30.43)	71 (35.50)	71 (35.86)	
≥80 years	61 (29.47)	47 (23.50)	42 (21.21)	
**Sex (%)**				
Male	137 (66.18)	99 (49.50)	121 (61.11)	0.002
Female	70 (33.82)	101 (50.50)	77 (38.89)	
**Race (%)**				
Mexican American	24 (11.60)	20 (10.00)	34 (17.18)	<0.001
Other Hispanic	27 (13.04)	19 (9.50)	31 (15.66)	
Non-Hispanic White	95 (45.89)	75 (37.50)	62 (31.31)	
Non-Hispanic Black	41 (19.81)	65 (32.5)	64 (32.81)	
Other race	20 (9.66)	21 (10.5)	7 (3.54)	
**Educational level (%)**				
Below high school	86 (41.55)	71 (35.50)	111 (56.06)	<0.001
High school	43 (20.77)	57 (28.50)	43 (21.72)	
Above high school	78 (37.68)	72 (36.00)	44 (22.22)	
**Marital status (%)**				
Married/living with partner	124 (59.90)	102 (51.00)	97 (48.99)	0.063
Widowed/divorced/separated/never married	83 (40.10)	98 (49.00)	101 (51.01)	
**Poverty–income ratio (%)**				
≤1	52 (25.12)	58 (29.00)	68 (34.34)	0.124
>1	155 (74.88)	142 (71.00)	130 (65.66)	
**Body mass index (%)**				
< 25 kg/m^2	61 (29.47)	59 (29.50)	43 (22.73)	0.241

25-30 kg/m^2	82 (39.61)	67 (33.50)	75 (38.38)	
≥ 30 kg/m^2	64 (30.92)	74 (37.00)	74 (38.89)	
**Smoking status (%)**				
Never	101 (48.79)	101 (50.50)	92 (46.46)	0.199
Former	89 (43.00)	76 (38.00)	75 (37.88)	
Current	17 (8.21)	23 (11.50)	31 (15.66)	
**Hypertension (%)**	141 (68.12)	152 (76.00)	138 (69.70)	0.180
**Diabetes (%)**	52 (25.12)	62 (31.00)	62 (31.31)	0.300
**Renal failure (%)**	18 (8.70)	18 (9.00)	24 (12.12)	0.447

*p value was tested by Chi-square test or Fisher’s exact

Table S2. Weighted odds ratios (95% confidence intervals) of low cognitive performance by quartiles of metals level, NHANES 2011–2014

	CERAD Test			Animal Fluency Test			DSST		
group	Model1	Model2	Model3	Model1	Model2	Model3	Model1	Model2	Model3
Urine-Mo (μg/g creatinine)									
Q1 (< 26.46)	reference	reference	reference	reference	reference	reference	reference	reference	reference
Q2 (≥ 26.46 & < 39.25)	0.714 (0.326-1.562)	0.598 (0.264-1.359)	0.573 (0.228-1.439)	1.086 (0.614-1.921)	0.960 (0.539-1.709)	0.963 (0.489- 1.896)	0.831 (0.377-1.830)	0.738 (0.331-1.645)	0.658 (0.241-1.795)
Q3 (≥ 39.25 & < 59.5)	0.667 (0.337-1.318)	0.693 (0.368-1.305)	0.692 (0.351-1.364)	0.697 (0.353-1.376)	0.675 (0.351-1.299)	0.664 (0.323-1.365)	0.866 (0.473-1.584)	0.895 (0.512-1.565)	0.912 (0.419-1.982)
Q4 (≥ 59.5)	0.988 (0.472-2.068)	0.995 (0.478-2.071)	0.931 (0.437-1.981)	0.830 (0.447-1.541)	0.779 (0.423-1.433)	0.693 (0.375-1.281)	0.537 (0.265-1.089)	0.510 (0.266-0.979)	0.459 (0.207-1.017)
Urine-Pb (μg/g creatinine)									
Q1 (< 0.3358)	reference	reference	reference	reference	reference	reference	reference	reference	reference
Q2 (≥ 0.3358 & < 0.5138)	1.179 (0.679-2.044)	1.180 (0.710-1.960)	1.364 (0.790-2.352)	1.033 (0.617-1.730)	1.018 (0.601-1.722)	1.244 (0.665- 2.326)	0.860 (0.455-1.626)	0.841 (0.453-1.562)	1.092 (0.511-2.336)
Q3 (≥ 0.5138 & < 0.7857)	1.291 (0.847-1.967)	1.137 (0.731-1.768)	1.336 (0.772-2.310)	0.861 (0.589-1.257)	0.728 (0.512-1.035)	0.877 (0.559-1.377)	0.680 (0.395-1.169)	0.581 (0.334-1.012)	0.677 (0.387-1.184)
Q4 (≥ 0.7857)	1.216 (0.689-2.146)	1.061 (0.620-1.817)	1.305 (0.705-2.418)	0.802 (0.445-1.445)	0.660 (0.367-1.185)	0.817 (0.389-1.714)	0.800 (0.420-1.522)	0.684 (0.354-1.320)	0.843 (0.371-1.917)
Urine-Sn (μg/g creatinine)									
Q1 (< 0.4174)	reference	reference	reference	reference	reference	reference	reference	reference	reference
Q2 (≥ 0.4174 & < 0.7457)	1.273 (0.672-2.413)	1.624 (0.840-3.139)	1.565 (0.805-3.043)	0.908 (0.540-1.527)	0.950 (0.541-1.669)	0.912 (0.521-1.597)	0.741 (0.376-1.460)	0.822 (0.407-1.662)	0.846 (0.393-1.820)
Q3 (≥ 0.7457 & < 1.7333)	0.919 (0.465-1.815)	1.093 (0.516-2.317)	1.002 (0.460-2.183)	0.884 (0.476-1.641)	0.793 (0.417-1.511)	0.697 (0.331-1.465)	0.802 (0.419-1.536)	0.842 (0.451-1.573)	0.743 (0.372-1.484)

Q4 (≥ 1.7333)	1.558 (0.840-2.890)	1.591 (0.817-3.099)	1.313 (0.615-2.804)	1.410 (0.787-2.524)	1.147 (0.640-2.057)	0.935 (0.500-1.749)	1.554 (0.843-2.867)	1.463 (0.813-2.632)	1.137 (0.604-2.141)
Urine-Ur (μg/g creatinine)									
Q1 (< 0.00377)	reference	reference	reference	reference	reference	reference	reference	reference	reference
Q2 (≥ 0.00377 & < 0.00648)	1.437 (0.780-2.647)	1.724 (0.906-3.281)	1.533 (0.734-3.203)	1.260 (0.749-2.120)	1.275 (0.764-2.127)	1.028 (0.608-1.737)	1.271 (0.654-2.471)	1.360 (0.701-2.638)	1.096 (0.533-2.254)
Q3 (≥ 0.00648 & < 0.01224)	2.085 (1.212-3.586)	2.745 (1.484-5.075)	2.541 (1.363-4.737)	1.091 (0.511-2.330)	1.034 (0.473-2.258)	0.806 (0.324-2.006)	0.824 (0.457-1.488)	0.846 (0.460-1.559)	0.562 (0.248-1.275)
Q4 (≥ 0.01224)	1.138 (0.529-2.450)	1.513 (0.731-3.133)	1.305 (0.594-2.869)	1.360 (0.720-2.569)	1.285 (0.676-2.441)	1.145 (0.557-2.351)	1.006 (0.485-2.087)	1.074 (0.550-2.096)	1.014 (0.447-2.297)

Table S2. Crude model (Model 1) did not adjust any confounders. Model 2 adjusted for age (years), gender. Model 3 was the same as Model 2 with additional adjustment for educational level (less than high school, high school, higher than high school), marital status (married, widowed, divorced, separated, never married, living with partner), BMI, PIR, smoke, race, renal failure (Yes or No), hypertension (Yes or No) and diabetes (Yes or No).
